# Accuracy of Digital Dental Implants Impression Taking with Intraoral Scanners Compared with Conventional Impression Techniques: A Systematic Review of In Vitro Studies

**DOI:** 10.3390/ijerph19042026

**Published:** 2022-02-11

**Authors:** María Isabel Albanchez-González, Jorge Cortés-Bretón Brinkmann, Jesús Peláez-Rico, Carlos López-Suárez, Verónica Rodríguez-Alonso, María Jesús Suárez-García

**Affiliations:** 1 Department of Conservative Dentistry and Orofacial Prosthodontics, Faculty of Dentistry, Complutense University of Madrid, 28040 Madrid, Spain; malbanch@ucm.es (M.I.A.-G.); jpelaezr@ucm.es (J.P.-R.); carlopezsuarez@gmail.com (C.L.-S.); veranicr@ucm.es (V.R.-A.); mjsuarez@ucm.es (M.J.S.-G.); 2 Department of Dental Clinical Specialties, Faculty of Dentistry, Complutense University of Madrid, 28040 Madrid, Spain

**Keywords:** accuracy, conventional impression, dental implant, digital impression, in vitro studies, systematic review

## Abstract

The aim of this systematic review was to evaluate the in vitro accuracy of dental implants impressions taken with intraoral scanner compared with impressions taken with conventional techniques. Two independent reviewers conducted a systematic electronic search in the PubMed, Web of Science and Scopus databases. Some of the employed key terms, combined with the help of Boolean operators, were: “dental implants”, “impression accuracy”, “digital impression” and “conventional impression”. Publication dates ranged from the earliest article available until 31 July 2021. A total of 26 articles fulfilled the inclusion criteria: 14 studies simulated complete edentation (CE), nine partial edentation (PE) and only two simulated a single implant (SI); One study simulated both CE and SI. In cases of PE and SI, most of the studies analyzed found greater accuracy with conventional impression (CI), although digital impression (DI) was also considered adequate. For CE the findings were inconclusive as six studies found greater accuracy with DI, five found better accuracy with CI and four found no differences. According to the results of this systematic review, DI is a valid alternative to CI for implants in PE and SI, although CI appear to be more accurate. For CE the findings were inconclusive, so more studies are needed before DI can be recommended for all implant-supported restorations.

## 1. Introduction

Passive fit between prosthetic structure and supporting implants is considered as the key factor in preventing subsequent mechanical and biological complications and, therefore, in the long-term success of implant-supported prosthodontic treatments. Among the mechanical complications, the tension, compression and flexion forces derived from poor passive fit can result in screw loosening or fracture, unfavorable movements, prosthesis breakage, or even implant fracture. Biological complications may also be caused by poor fit due to the gap between the prosthesis and the implant, that hosts an accumulation of microorganisms causing biological problems in the supporting tissues [1,2].

Various authors have pointed out that obtaining absolute passive fit is almost impossible due to the number of steps involved in the prosthetic fabrication process, especially in full-arch and partial restorations, supported by several implants [3,4]. Nevertheless, there is a small margin of error that can be tolerated without occasioning future complications [2,5]. So passive fit is defined as the level of fit that does not lead to long-term clinical complications [6]. While some authors claim that the maximum acceptable lack of fit is 150 µm [6], others place the limit at 60 µm [2]. Others argue that implants present a maximum mobility of 50 µm in bone [7], so this value can be considered the maximum lack of fit for each implant/restoration complex [8].

The advent of Computer-aided design/computer-aided manufacturing (CAD/CAM) systems has been accompanied by the introduction of digital impression (DI) using intraoral scanners (IOSs) within a fully digital workflow. DIs are taken by IOSs, which, such as an ordinary camera, collect information about projecting light. Reproducible tissues are shown on the hardware display as natural looking. Intraoral cameras use video technique or still photo technique for scanning [9]. DIs bring certain advantages including lower risk of distortion during the impression taking and model fabrication and increased patient comfort [10,11,12].

Both early and most recent systematic reviews analyzing marginal fit of fixed dental prosthesis (FDPs) manufactured from CIs versus DIs show greater marginal accuracy in DIs group [13,14]. However, few studies have compared these impressions in implants, leading to a lack of consensus about which of these two techniques is more accurate.

The objective of this systematic review was to evaluate and compare the in vitro accuracy of dental implants impressions taken with IOSs compared with impressions taken with conventional techniques. As far as the authors know, this is the first attempt to summarize exclusively in vitro studies that compare accuracy of CI and DI of dental implants. The null hypothesis was that there is no difference between CIs and DIs of dental implants in terms of impression accuracy.

## 2. Materials and Methods

### 2.1. Protocol Development and PICO Question

Two independent reviewers (MIAG and JCBB) conducted a systematic literature search following PRISMA (Preferred Reporting Items for Systematic Reviews and Meta-Analyses) guidelines (see PRISMA checklists in Supplmentary Materials in [15]). The review was not registered. The PICO (Population, Intervention, Comparison, Outcome) question was: “Are DIs taken with IOS more accurate than CIs for implant-supported prostheses?”

- Population: edentulous and partially edentulous patients in need of implant-supported prostheses.
- Intervention: DI taken with IOS.
- Control: CI.
- Outcome: accuracy of impressions.

### 2.2. Search Strategy

The systematic search was conducted in the electronic databases PubMed, Web of Science and Scopus. The MeSH terms and the search strategy according to the focused PICO question are presented in Table 1. No limits were placed on publication date, so the review included reports dated from the first available article until 31 July 2021. Inter-reviewer reliability was assessed obtaining a Kappa coefficient of 0.74 after the selection by title and 0.85 after the full-text reading of the articles selected by abstract, in which values above 0.8 are considered a good level of agreement [16]. When disagreement arose between the two main reviewers (MIAG and JCBB), it was resolved through discussion and consensus between them and a third reviewer (MJSG).

**Figure 1:**
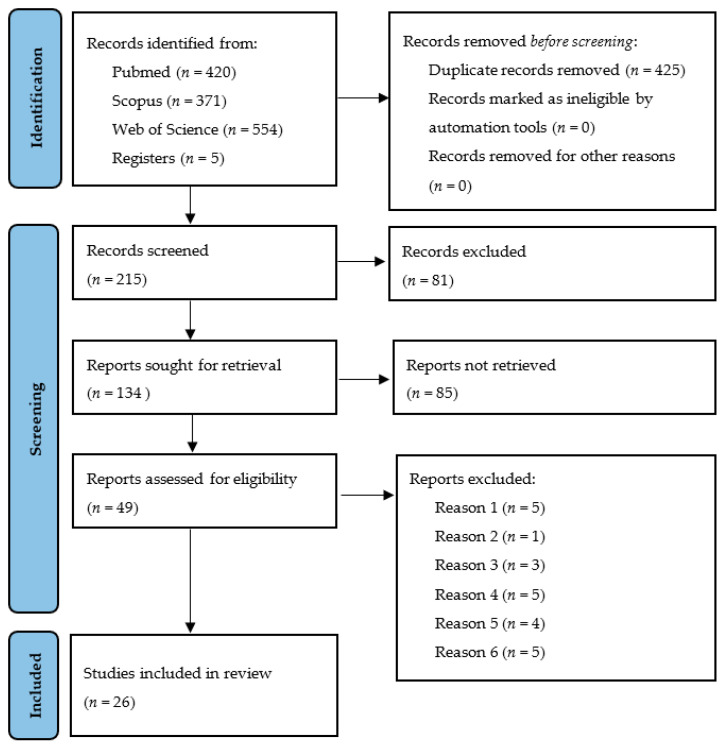
PRISMA flow chart diagram.

The first selection of articles was based on titles and abstracts, and a second selection was performed after reading the full text (Figure 1). Initial electronic search was complimented by manual search in the references section of the selected articles after the full text reading. Manual search was also performed in the next relevant journals: Journal of Dental Research, Clinical Oral Implants Research, Journal of Dentistry, Clinical Implant Dentistry and Related Research, Journal of Prosthetic Dentistry, Journal of Prosthodontic Research, European Journal of Oral Implantology, Journal of Prosthodontics, International Journal of Oral and Maxillofacial Implants, International Journal of Prosthodontics, Quintessence International, Journal of Evidence-Based Dental Practice, Implant Dentistry, International Journal of Computerized Dentistry and Journal of Oral Implantology.

### 2.3. Inclusion Criteria

Inclusion criteria were as follows:

- Studies in vitro as only in vitro research can directly compare the accuracy of two impression techniques.
- Studies that test impressions of dental implants in cases of complete edentation (CE), partial edentation (PE) and/or single implant (SI).
- Studies that compare one or various CI techniques versus DI using one or various IOSs. In other words, studies had to make a direct comparison of these two types of impressions.
- Studies that evaluate the accuracy (trueness, precision, or both) of the compared impression techniques, and provide complete information about the methods.
- Studies published in peer reviewed journals and in English.

### 2.4. Exclusion Criteria

In vivo studies, clinical case reports, articles about single techniques and articles that did not match the objectives of the present review were excluded. The studies excluded at the second selection stage (after full-text reading) and the reasons for exclusion are summarized in Table 2.

### 2.5. Data Extraction and Quality Evaluation

Descriptive analysis of the selected articles was performed. The extracted data was:

- Study design.
- Type of edentation.
- Number of implants.
- Implant angulation.
- Implant brand/model.
- Impression techniques compared.
- Sample size (number of impressions taken in each type of analyzed techniques).
- IOS used.
- Method of evaluation of the accuracy.
- Main accuracy results.

The parameter to analyze and compare was the accuracy of the impression techniques. Accuracy is the key parameter to ensure passive fit between implants and the prosthetic structure and is defined by two parameters (ISO 5725-1) [39]:

- Trueness: the impression’s proximity to measured reality, it is the main concept associated with accuracy.
- Precision or reproducibility: similarity between a group of impressions of the same case.

It was not possible to perform statistical analysis to synthesize the results of the different studies due to the different methods employed.

As clinical trials were not included in the review, quality evaluation such as CASP (Critical Appraisal Skills Program) or similar could not be made. The CONSORT (Consolidated Standards of Reporting Trials) checklist adapted to in vitro studies of dental materials by Flaggion et al. [40] was used to assess the quality of the articles; The authors of the present review further adapted this checklist for studies that compare the accuracy of dental implants impression techniques: items from 5 to 9 were eliminated due to selected studies are in vitro, so there is a low risk of bias, no need for randomization and sample size is not so determining (Table A1). Only studies obtaining a score of 80% or more were included in the present review (Table A2).

## 3. Results

### 3.1. Study Selection

The initial electronic search identified 425 articles after removing the duplicates. On the basis of the title alone, 215 articles were selected, of which 49 were selected on the basis of the abstract. After reading the full text, 26 articles fulfilled the inclusion criteria (Figure 1).

All the articles included in the review were in vitro studies (Table 3). Although Alshabarty et al. [41] described itself as an in vivo study, it could be classified as in vitro because the reference models used, although they proceeded from impressions taken in patients, were effectively in vitro models. In this case, 14 articles investigated cases of CE exclusively [11,42,43,44,45,46,47,48,49,50,51,52,53,54], nine of PE [41,55,56,57,58,59,60,61,62], two of SI [63,64] and another included both CE and SI models [65].

### 3.2. Study Characteristics

The information extracted from the articles, included the number of implants, presence or not of angulation or abutments and other data are summarized in Table 3. The excluded studies and the reasons for exclusion are summarized in Table 2.

The parameter to analyze and compare was the accuracy of the impression techniques (trueness and precision): to evaluate and compare the trueness of different impressions, the intraoral position of the dental implants must be reproduced by a high precision instrument to obtain a reference model [66]. However, because of the anatomical characteristics of the oral cavity, this reproduction cannot be performed by high precision instruments such as a coordinate measurement machine (CMM) or an extraoral laboratory scanner, which is the reason why in vivo studies cannot compare trueness of different impression techniques and were excluded from the present review. In vitro studies include reference models and test models measured by the same high precision instruments (CMM, microscopes, etc.) and so trueness can be measured by determining the deviation between these two sets of models [66]. However, in vivo studies can only analyze the trueness of impressions indirectly, for example, by analyzing the fit of prosthetic structures fabricated via CI versus DI, which adds steps to the analysis process and gives more information about the feasibility of the impression than on its accuracy itself.

The methods used to analyze the accuracy of the impressions in the reviewed studies were diverse and included the following:

- Analysis of prosthetic fit, examining marginal gaps with an optical microscope.
- Analysis of deviations in the distance between implants with digital calipers.
- Analysis of linear, angular and/or three-dimensional deviation by superimposing models as STL (surface tessellation language) files using softwares such as Geomagic Control X (3D systems, Rock Hill, SC, USA). Conventional plaster models are transformed into STL files by scanning them with an extraoral laboratory scanner. Best-fit algorithm is one of the most common methodologies used to investigate accuracy through STL superimposition. Other techniques include the “least squares method” or the “zero method”.
- Analysis of linear, angular and/or three-dimensional deviations with a CMM and the corresponding metrological software.

Most of the studies used the two latter methods listed above.

Results of quality assessment are summarized in Table A2. According to the modified CONSORT checklist used, five articles obtained a score of 90% and the rest 100%.

### 3.3. CE Studies

Of the 15 in vitro studies that simulated CE, six reported greater accuracy with DI [43,44,45,50,52,65], four found no difference [42,46,48,53] and five reported greater accuracy with CI [11,47,49,51,54].

Two of these studies used similar methods [42,43] employing master models with five identical implants with angulations of 0°, 10° and 15° and comparing open tray CI technique with splinting and DI. They analyzed accuracy by superimposing STL files. Papaspyridakos et al. [42] found no differences between the techniques but Amin et al. [43] found greater accuracy with DI. Neither article reported that impressions accuracy was significantly affected by implant angulation.

Abdel-Azim et al. [65] used a master model with four parallel implants and compared closed tray CI with DI, performed at abutment level. Accuracy was analyzed by observing the marginal gap under a microscope between restorations fabricated using digital and conventional workflows. DI obtained greater accuracy.

Menini et al. [44] also took impressions at abutment level, but compared three-dimensional deviations by CMM, finding DI to be more accurate.

Alikhasi et al. [45] compared CI and DI in implants with internal and external connections. Three-dimensional deviations were measured with CMM, finding DI to be more accurate and unaffected by angulation or connection type.

Taking the most recent studies [11,46,47,48,49,50,51,52,53,54], no consensus could be construed from the results as three did not find differences between DI and CI [46,48,53], two found greater accuracy of DI [50,52] and five found greater accuracy of CI [11,47,49,51,54]. Of this latter five articles, three used similar methods [11,47,49] in terms of impression taking techniques and accuracy evaluation (CMM). Of the three studies that did not find differences between CI and DI, two took CIs without splinting [48,53].

Regarding implant angulation, three of the five CE studies that analyzed the effect of angulation in both DI and CI found that angulation did not affect any of them. Alikhasi et al. [45] found that CI are affected by angulation while DI not, and Ribeiro et al. [50] that DI had better accuracy only in parallel implants, while there were no differences between CI and DI in angulated implants.

Concerning the impression level (abutment vs. implant-level), CE studies are the only group of studies that includes both studies with abutment-level and implant-level impressions, while all the PE and SI studies performed implant-level impressions. Comparing the results of CE studies that performed implant-level impressions with those that performed abutment-level impressions, no differences were observed since both present the same proportion of studies that showed greater accuracy of DIs, CIs or that did not find differences between them.

### 3.4. PE Studies

Among the nine studies that simulated PE, all used two implants, with the exception of one study with three implants [61]. Seven reported greater accuracy with CI [41,55,56,57,58,60,61] and only two found greater accuracy with DI [59,62]. It should be noted that Chew et al. [57] found greater accuracy with CI for bone level implants but not for tissue level implants.

Regarding implant angulation, of the four PE studies that included implants with varying angulation, no consensus can be found: one reported that angulation had an influence on the accuracy of DI [55], another that it influenced accuracy of CI but not DI [62], another that it influenced both techniques [58] and the other did not observe any influence on either technique [56].

### 3.5. SI Studies

The two oldest studies that simulated SI [63,65] found greater accuracy with CI. Both compared DI and CI with closed tray, but one took impressions at implant level [63] and the other at abutment level [65].

Lee et al. [63] reported worse reproduction of secondary anatomy, sulci, and fossae with DI. Regarding the vertical position of the implants, it was seen that in DI it tended to be more coronal, which would produce a lack of contact of the final restoration, and more apical in CI (due to the elasticity of the material), which would occasion premature contacts. The latter circumstance would be more favorable as it could be corrected clinically, while a lack of contact could not.

Nevertheless, the most recent study for SI found greater accuracy with DI than with open-tray implant-level CI [64].

## 4. Discussion

This systematic review was set out to evaluate the accuracy of IOSs for dental implants impressions compared with conventional techniques. The data extracted from the articles were limited by the fact that the review only included in vitro studies. The main obstacle to performing in vivo studies of impression taking is the lack of an established protocol for evaluating the accuracy of intraoral impressions. The positions of implants in the mouth must be reproduced by a high precision instrument to obtain a reference model, but because of the anatomical characteristics of the oral cavity this reproduction cannot be realized by high precision instruments such as a CMM or an extraoral laboratory scanner. However, in vitro studies include reference models and test models measured by these instruments. In this way, trueness can be measured in terms of deviation between reference and test models [66]. Nevertheless, since reference optical scanners cannot be used intraorally, in vivo studies only can analyze trueness indirectly by Sheffield testing and microscopic and/or radiographic evaluation of prosthetic structures fabricated from CIs or DIs [52]. Although in vivo studies are not able to compare trueness, they can measure precision (reproducibility) by evaluating the deviations among a group of impressions taken with the same technique within a single patient. In this sense, Mühlemann et al. [67] compared the precision of DI and CI (closed tray impressions at implant level) by means of superimposing STL files in five patients with SIs of the same brand in the posterior region; CI was found to obtain greater precision.

Another limitation is the lack of homogeneity among the included studies in terms of factors that can directly affect the accuracy of impressions such as the IOS working principle, the scanning strategy, the scanned area, the splinting or not of CIs, the presence or not of implants abutments, etc. In addition, there are differences in methodology regarding methods of data analysis, data selection and impressions accuracy analysis and comparison.

Lack of homogeneity among the studies made it difficult to reach clear conclusions, especially for implant-supported restorations in CE patients, in which the 15 CE studies used obtained diverse results. According to six of these studies, DI was found to be more accurate [43,44,45,50,52,65], while four articles did not find differences between the techniques [42,46,48,53], and five found greater accuracy with CI [11,47,49,51,54]. One of the reports that observed greater accuracy with DI [65] performed closed tray CI which, according to the systematic review by Papaspyridakos et al. [68] is less accurate compared with open tray technique. Moreover, the study compared CAD/CAM milled restorations with conventional cast metal restorations. It should also be noted that among the four studies that found no differences between DI and CI, one took impressions at abutment level [46] and the other two took not-splinted CIs [48,53]. The studies by Papaspyridakos et al. [42] and Amin et al. [43] employed similar methods; the fact that their results (the former found no differences while the latter found greater accuracy with DI) could be attributed to various factors: the IOSs and scan bodies used were different and in Papaspyridakos et al. [42] the procedures were carried out by experienced clinicians while in Amin et al. [43] impressions were carried out by interns of medium-term experience. Regarding the experience factor, while Lee et al. [34] reported greater efficiency of DI realized by inexperienced students, studies by Gimenez et al. [69,70,71] did not reach conclusive results about this factor.

The different in vitro and in vivo studies that analyze the viability of DI in cases of CE show that IOS may be apt in these cases [25,72,73,74]. This reflects the improvements in IOSs over the years as less recent studies [8,71] reported they were not valid, partly due to the accumulation of error caused by the lack of anatomical references for linking images. It should be noted that the overlapping failures produced in the first in vivo studies of DI of implants in CE [8] were not observed in subsequent in vitro studies, even when the same IOS model was used [55,63,65,69,75]. To overcome the lack of intraoral features available for overlapping images in cases of CE, various solutions have been proposed: splinting scanbodies [12], adding landmarks to the residual ridge [76] or using scan bodies with elongated extensions [51].

Despite the heterogeneity of the studies and the impossibility of making in vitro and in vivo studies comparable, Papaspyridakos et al. [77] recently published a systematic review and meta-analysis comparing digital and conventional implant impressions including both in vitro and in vivo studies. However, we must take their results into account since most in vitro studies selected coincide with those of the present review. For CE impressions they found nominally less deviation for DI but no statistically significant differences. Nonetheless, a prosthesis prototype try-in before the fabrication of the definitive prosthesis is still recommended for complete digital workflows of CE cases.

For simulated PE, all studies analyzed except two [59,62] reported greater accuracy of CI [41,55,56,57,58,60,61]. One study found greater accuracy with CI of bone level implants but no differences between CI and DI of tissue level implants [57]. Another report found greater accuracy with CI for parallel implants but not for angulated implants [58]. Although the number of analyzed studies was insufficient to reach significant conclusions, it was seen that in cases of PE at the present time, CIs obtain greater accuracy. For SI, the two oldest studies that simulated SI [63,65] found greater accuracy with CI. Nevertheless, the most recent study for SI found better accuracy with DI than with CIs taken with open tray and implant-level [64].

Numerous authors have observed the decreasing accuracy of IOSs as the area scanned increases [9,24,33,73,78,79,80,81,82,83]. In this way, one of the factors cited as limiting the accuracy of DI is the image processing software used, which joins images together and projects the implant position on the basis of scan body images [71]. It should be noted that cases of CE involve scanning larger areas and more image superimposition than cases of PE. Consequently, DIs are considered clinically safer and more appropriate for cases of PE than CE, although the in vitro studies included in the present review find greater accuracy with CI for PE. In the systematic review and meta-analysis of Papaspyridakos et al. [77] CI had nominally less deviation than DI for PE impressions, with statistically significant differences.

Regarding the impact of the impression level (abutment vs. implant-level) on the accuracy, this parameter could only be analyzed in the group of CE studies since this group included both studies with abutment-level and implant-level impressions, while all the PE and SI studies performed exclusively implant-level impressions. In this context comparing the results of CE studies that performed implant-level impressions with those that performed abutment-level impressions, no differences were observed since both groups present the same proportion of studies that showed greater accuracy either of DIs or CIs. Therefore, it appears that the impression level did not influence the results of the analyzed impressions accuracy. However, we must consider the inherent methodological differences between the different studies. Papaspyridakos et al. study [42] was the only one that includes both abutment-level and implant-level CIs in the same study, observing greater accuracy of DIs compared to non-splinted implant-level CIs, while no differences were observed between DIs and splinted implant-level, splinted abutment-level and non-splinted abutment-level CIs. Therefore, performing implant-level impressions only influenced the non-splinted group.

Concerning implants angulation, nine studies analyzed the effect of implant angulation on the accuracy of impression taking. One affirmed that angulation exerted an influence on both impression techniques [58], two reported that angulation only affected DI [50,55] and other two found that angulation affected CI [45,62]. The rest of the studies found that implant angulation had no influence on impression accuracy [42,43,46,56], a finding that concurs with studies by Gimenez et al. [69,70,71], who did not observe that angulation affected the accuracy of DI carried out with different scanners. The systematic review published by Flügge et al. [12] in 2019 presents evidence that CI is less exact when taking impressions of angulated implants, while DI showed no differences between angulated and parallel implants.

An important limitation on the present review was the small number of studies that have analyzed and compared the accuracy of DI and CI of implants, which points to the need for further in vitro studies of the topic. The studies published to date used very contrasting methods, so it is necessary to establish a consensual protocol for analyzing the accuracy of impressions techniques in order to make reliable comparisons of the obtained results. This need has already been highlighted in systematic reviews by Alikhasi et al. [84] and Flügge et al. [12].

## 5. Conclusions

For implant-supported restorations in cases of PE and SI, the scant evidence available suggests that CI is more accurate. However, due to the lesser image superimposition required and the smaller distances between the implants, IOSs are also considered suitable.

For implant-supported restorations in cases of CE, there is insufficient evidence to reach firm conclusions. Nevertheless, most recent studies have reported they may be used effectively in cases of CE, and some studies have found DI to be more accurate.

Further studies are needed, with more rigorous and consensual methods. IOSs require improvement so that DI may be used with confidence in all cases requiring implant-supported prostheses.

## Figures and Tables

**Table 1:** Search strategy according to the focused question (PICO).

Focused Question (PICO)	Are DIs Taken with IOS More Accurate than CIs for Implant-Supported Prostheses?	
Search strategy	Population	Edentulous and partially edentulous patients in need of implant-supported prostheses. #1-((dental implants [MeSH]) OR (edentulous patients [MeSH]) OR (single implant [MeSH]) OR (multiple implants [MeSH]) OR (partially edentulous arch [MeSH]) OR (complete arch [MeSH]) OR (full arch [MeSH]))
	Intervention	Digital impression (DI) with intraoral scanner (IOS) #2-((digital impression [MeSH]) OR (intraoral scanner [MeSH]) OR (dental scanner [MeSH]) OR (implant impressions [MeSH]) OR (impression making [MeSH]) OR (implant rehabilitation [MeSH]) OR (implant restoration [MeSH]) OR (digital techniques [MeSH]))
	Comparison	Conventional impression (CI) #3-((conventional impression [MeSH]) OR (traditional impression MeSH]) OR (open-tray impression [MeSH]) OR (closed-tray impression [MeSH]) OR (conventional techniques [MeSH]))
	Outcome	Accuracy of impressions #4-((impression accuracy [MeSH]) OR (trueness [MeSH]) OR (precision [MeSH]) OR (in vitro study [MeSH]))
	Search combination(s)	(#1) AND (#2) AND (#3) AND (#4)

**Table 2:** Studies excluded after reading the full text and reasons for exclusion.

Studies	Reason for Exclusion
Eliasson et al., 2012 [17]; Howell et al., 2013 [18]; Al-Abdullah et al., 2013 [19]; Ng et al., 2014 [20]; Ajioka et al., 2016 [21]	Use of healing abutments instead of scan-bodies
Karl et al., 2012 [22]	Use of scannable cementable abutments instead of scan-bodies
Mangano et al., 2016 [23]; Imburgia et al., 2017 [24]; Pesce et al., 2018 [25]	No comparison between DI and CI, only between different IOS
Stimmelmayr et al., 2012 [26]; Ono et al., 2013 [27]; Bergin et al., 2013 [28]; Stimmelmayr et al., 2013 [29]; Jokstad et al., 2015 [30]	Use of extraoral scanner, not IOS
Andriessen et al., 2014 [8]; Rhee et al., 2015 [31]; Gedrimiene et al., 2019 [32]; Chochlidakis et al., 2020 [33]	No comparison between reference model and test model, only between two different test models
Lee et al., 2013 [34]; Wismeijer et al., 2014 [35]; Joda et al., 2015 [36]; Schepke et al., 2015 [37]; Joda et al., 2017 [38]	Evaluate efficiency of scanning, scanning learning curve or patient preference.

**Table 3:** Summary of selected studies.

Author and Year	Edentation	Number of Implants	Angulation	Implants Label	Impression Techniques	Sample Size	IOS	Assessment of Accuracy	Outcomes (Greater Accuracy)
Abdel-Azim et al., 2014 [65]	CE and SI	4, 1	--	Straumann TL	Closed tray (abutment level) DI (abutment level)	6	iTero	Marginal discrepancy (microscopy)	SI: CI CE: DI
Papaspyridakos et al., 2016 [42]	CE	5	0°, 10°, 15°	Straumann BL	Open tray (with and without splinting, abutment and implant level) DI (implant level)	10	Trios (3Shape)	3D deviation (stl superimposition)	No differences (DI more accurate than non-splinted implant level) Angulation did not affect any of them
Amin et al., 2017 [43]	CE	5	0°, 10°, 15°	Straumann BL	Open tray (splinted, implant level) DI (implant level)	10	CEREC Omnicam 4.4.1 (Sirona) True Definition 4.1 (3M ESPE)	3D deviation (stl superimposition)	DI True Definition greater accuracy Angulation did not affect any of them
Menini et al., 2018 [44]	CE	4	--	Biomet 3i	Open tray (with and without splinting, abutment level) Closed tray (abutment level) DI (abutment level)	5	True Definition (3M ESPE)	Linear and angular deviation (CMM) Marginal discrepancy (Sheffield test, microscopy)	DI
Alikhasi et al., 2018 [45]	CE	4	0°, 45°	Nobel Replace Nobel Branemark	Open tray (non-splinted, implant level) Closed tray (implant level) DI (implant level)	15	Trios (3Shape)	Linear and angular deviation (CMM)	DI (no affected by platform or angulation)
Gintaute et al., 2018 [53]	CE	2, 4, 6	0°, 45°	Biomet 3i Certain	Open tray (non-splinted, implant level) DI (implant level)	5	True Definition (3M ESPE)	3D deviation (CMM)	No differences
Moura et al., 2019 [46]	CE	6	0°, 15°	Implacil (external connection)	Open tray (splinted, abutment level) Closed tray (abutment level) DI (abutment level)	5	Dental Wings 3 series (Straumann)	Digital caliper (linear deviation)	No differences Angulation did not affect any of them
Kim et al., 2019 [47]	CE	5	--	Warantec IU	Open tray (splinted, implant level) DI (implant level)	10	Trios 3 (3Shape)	Linear and angular deviation (CMM)	CI
Rech-Ortega et al., 2019 [48]	CE	6	--	Biomet 3i Certain	Open tray (non-splinted, implant level) DI (implant level)	20	True Definition (3M ESPE)	Linear and angular deviation (CMM)	No differences
Tan et al., 2019 [49]	CE	6, 8	--	Straumann BL	Open tray (splinted, implant level) DI (implant level)	5	Trios True Definition	Linear, angular and 3D deviation (CMM)	CI TRIOS greater accuracy
Ribeiro et al., 2019 [50]	CE	4	0°, 15°	Klockner KL	Closed tray (implant level) Open tray (with and without splinting, implant level) DI (implant level)	10	True Definition (3M ESPE)	3D deviation (stl superimposition)	DI (in parallel implants) Angulated implants: no differences
Huang et al., 2020 [51]	CE	4	--	Straumann BL	Open tray (splinted, abutment level) DI (abutment level; 3 different scanbody designs)	10	Trios 3 (3Shape)	3D deviation (stl superimposition)	CI
Albayrak et al., 2020 [52]	CE	8	40°, 20°, 25°, 15°	Dyna Helix DC	Open tray (non-splinted, abutment level) DI (abutment level)	10	Carestream 3500 Trios 3 Cerec Omnicam	Linear and angular deviation (reverse engineering software)	DI Carestream 3500 greater trueness
Revilla-León et al., 2020 [11]	CE	6	0°, 4°, 10°	Straumann BL	Open tray (splinted, abutment level) DI with IOS (abutment level) DI with photogrammetry (abutment level)	10	iTero (Cadent) Trios 3 (3Shape)	Linear, angular and 3D deviation (CMM)	CI Photogrammetry the least accurate
Lyu et al., 2021 [54]	CE	8	--	Camlog screw-line	Open tray (splinted, implant level) DI (implant level)	10	Trios 2 (3Shape)	Linear and 3D deviation (stl superimposition)	CI
Lin et al., 2015 [55]	PE	2	0°, 15°, 30°, 45°	Straumann TL	Open tray (non-splinted, implant level) DI (implant level)	10	iTero (Cadent)	Linear and angular deviation (stl superimposition)	CI DI: better behavior at higher angulation
Basaki et al., 2017 [56]	PE	2	0°, 10°, 30°	Straumann BL	Open tray (non-splinted, implant level) DI (implant level)	10	iTero (Cadent)	Linear and angular deviation (stl superimposition)	CI Angulation did not affect any of them
Chew et al., 2017 [57]	PE	2	--	Straumann BL Straumann TL	Open tray (non-splinted, implant level) DI (implant level)	5	Trios iTero True Definition	Linear and angular deviation (CMM)	CI (in BL) TL no differences TRIOS greater accuracy
Chia et al., 2017 [58]	PE	2	0°, 10°, 20°	Straumann BL	Open tray (non-splinted, implant level) DI (implant level)	5	Trios (3Shape)	Linear, angular and 3D deviation (CMM)	CI (in parallel implants) No differences in angulated implants Angulation affected both impressions
Marghalini et al., 2018 [59]	PE	2	30°	Nobel Replace Straumann TL	Open tray (splinted, implant level) DI (implant level)	10	Cerec Omnicam True Definition	3D deviation (stl superimposition)	DI True Definition greater accuracy
Alshawaf et al., 2018 [60]	PE	2	30°	Nobel Replace	Open tray (splinted, implant level) DI (implant level)	10	Cerec Omnicam True Definition	3D deviation (stl superimposition)	CI CEREC Omnicam greater accuracy
Bohner et al., 2019 [61]	PE	3	--	S.I.N Implant System	Open tray (splinted, implant level) DI (implant level)	10	Dental Wings (Straumann)	3D deviation (stl superimposition)	CI (in cusps)
Alsharbaty et al., 2019 [41]	PE	2	--	Implantium internal connection	Open tray (splinted, implant level) Closed tray (implant level) DI (implant level)	36	Trios 3 (3Shape)	Linear and angular deviation, interimplant distances (CMM)	CI
Abduo et al., 2021 [62]	PE	2	0°, 15°	Straumann TL	Open tray (splinted, implant level) Closed tray (non-splinted, implant level) DI (implant level)	10	Trios 4 Medit i500 True Definition	Linear and angular deviation (stl superimposition)	DI DI less affected by angulation True Definition less accuracy
Lee et al., 2015 [63]	SI	1	--	Straumann BL	Closed tray (implant level) DI (implant level)	30	iTero (Cadent)	3D deviation (stl superimposition)	CI (most favorable implant vertical position)
Yilmaz et al., 2021 [64]	SI	1	--	Neoss Proactive Straight	Open tray (implant level) DI with scan body (implant level) DI with healing abutment-scanpeg system (implant level)	10	Trios 3 (3Shape)	Linear and angular deviation (stl superimposition)	DI

CE: complete edentation. PE: partial edentation. SI: single implant. TL: tissue level. BL: bone level. CMM: coordinate measure machine. STL: standard tessellation language.

## Data Availability

The data presented in this study are available on request from the corresponding author.

## Appendix A

**Table A1:** Modified CONSORT checklist for in vitro studies comparing different dental implant impression techniques.

Section	Checklist Item
Abstract	Item 1. Structured summary of trial design, methods, results, and conclusions
Introduction	Item 2a. Scientific background and explanation of rationale
	Item 2b. Specific objectives and/or hypotheses
Methods	Item 3. The intervention for each group with sufficient detail to enable replication
	Item 4. Completely defined measures of outcome, including how and when they were assessed
	Item 5. Statistical methods used to compare groups for primary and secondary outcomes
Results	Item 6. For each primary and secondary outcome, results for each group, and the estimated size of the effect and its precision
Discussion	Item 7. Trial limitations, addressing sources of potential bias, imprecision and, if relevant, multiplicity of analyses
Other information	Item 8. Sources of funding and other support
	Item 9. Where the full trial protocol can be accessed, if available

**Table A2:** Results of the quality assessment of selected articles using the modified CONSORT checklist for in vitro studies comparing different dental implant impression techniques.

Author	Abstract	Introduction		Methods			Results	Discussion	Other		Result
	1	2a	2b	3	4	5	6	7	8	9	
Abdel-Azim et al., 2014 [65]	YES	YES	YES	YES	YES	YES	YES	YES	YES	YES	10/10
Papaspyridakos et al., 2016 [42]	YES	YES	YES	YES	YES	YES	YES	YES	YES	YES	10/10
Amin et al., 2017 [43]	YES	YES	YES	YES	YES	YES	YES	YES	YES	YES	10/10
Menini et al., 2018 [44]	YES	YES	YES	YES	YES	YES	YES	YES	YES	YES	10/10
Alikhasi et al., 2018 [45]	YES	YES	YES	YES	NO	YES	YES	YES	YES	YES	9/10
Gintaute et al., 2018 [53]	YES	YES	YES	YES	YES	YES	YES	YES	YES	YES	10/10
Moura et al., 2019 [46]	YES	YES	YES	YES	NO	YES	YES	YES	YES	YES	9/10
Kim et al., 2019 [47]	YES	YES	YES	YES	YES	YES	YES	YES	YES	YES	10/10
Rech-Ortega et al., 2019 [48]	YES	YES	YES	YES	YES	YES	YES	YES	YES	YES	10/10
Tan et al., 2019 [49]	YES	YES	YES	YES	YES	YES	YES	YES	YES	YES	10/10
Ribeiro et al., 2019 [50]	YES	YES	YES	YES	YES	YES	YES	YES	YES	YES	10/10
Huang et al., 2020 [51]	YES	YES	YES	YES	YES	YES	YES	YES	YES	YES	10/10
Albayrak et al., 2020 [52]	YES	YES	YES	YES	NO	YES	YES	YES	YES	YES	9/10
Revilla-León et al., 2020 [11]	YES	YES	YES	YES	YES	YES	YES	YES	YES	YES	10/10
Lyu et al., 2021 [54]	YES	YES	YES	YES	YES	YES	YES	YES	YES	YES	10/10
Lin et al., 2015 [55]	YES	YES	YES	YES	NO	YES	YES	YES	YES	YES	9/10
Basaki et al., 2017 [56]	YES	YES	YES	YES	YES	YES	YES	YES	YES	YES	10/10
Chew et al., 2017 [57]	YES	YES	YES	YES	YES	YES	YES	YES	YES	YES	10/10
Chia et al., 2017 [58]	YES	YES	YES	YES	YES	YES	YES	YES	YES	YES	10/10
Marghalini et al., 2018 [59]	YES	YES	YES	YES	YES	YES	YES	YES	YES	YES	10/10
Alshawaf et al., 2018 [60]	YES	YES	YES	YES	NO	YES	YES	YES	YES	YES	9/10
Bohner et al., 2019 [61]	YES	YES	YES	YES	YES	YES	YES	YES	YES	YES	10/10
Alsharbaty et al., 2019 [41]	YES	YES	YES	YES	NO	YES	YES	YES	YES	YES	9/10
Abduo et al., 2021 [62]	YES	YES	YES	YES	NO	YES	YES	YES	YES	YES	9/10
Lee et al., 2015 [63]	YES	YES	YES	YES	YES	YES	YES	YES	YES	YES	10/10
Yilmaz et al., 2021 [64]	YES	YES	YES	YES	YES	YES	YES	YES	YES	YES	10/10
